# The burden of familial chylomicronemia syndrome in Canadian patients

**DOI:** 10.1186/s12944-020-01302-x

**Published:** 2020-06-02

**Authors:** Daniel Gaudet, Michael Stevenson, Nelly Komari, Grace Trentin, Caroline Crowson, Nandini Hadker, Sophie Bernard

**Affiliations:** 1 Clinical Lipidology Unit, Department of Medicine, Université de Montréal, Chicoutimi, QC Canada; 2 ECOGENE-21 Clinical and Translational Research Center, Department of Medicine, Université de Montréal, 350 Jacques-Cartier B210, Chicoutimi, Québec G7H 7P2 Canada; 3 Akcea Therapeutics, 22 Boston Wharf Rd, Boston, MA 02210 USA; 4 Akcea Therapeutics, 1220-55 Metcalfe Street, Ottawa, ON K1P 6L5 Canada; 5 TRINITY, 230 3rd Avenue, Waltham, MA 02451 USA; 6 Lipids, Nutrition and Cardiovascular Prevention Clinic, Montreal Clinical Research Institute, 110 Avenue des Pins Ouest, Montréal, QC, H2W 1R7 Canada; 7 Department of Medicine, Division of Endocrinology, Université de Montreal, Montréal, QC Canada

**Keywords:** Familial chylomicronemia syndrome, Burden of illness, Acute pancreatitis, Quality of life, Lipoprotein lipase deficiency, Pancreatitis, Hyperlipoproteinemia, Hypertriglyceridemia, Canada, Founder effect

## Abstract

**Background:**

Familial chylomicronemia syndrome (FCS) is a rare autosomal recessive disorder characterized by persistent extreme hypertriglyceridemia as a result of lipoprotein lipase deficiency. Canada is an important region for FCS research due to the high prevalence rates. The burden of illness and quality of life of Canadian patients, however, have been inadequately addressed in the literature.

**Objective:**

To understand the burden of illness of FCS on Canadian patients’ lives.

**Methods:**

IN-FOCUS is a global web-based survey open to patients with FCS, including patients in Canada. This survey captured information on diagnostic experience, symptoms, comorbidities, disease management, and impact on multiple life dimensions.

**Results:**

A total of 37 Canadian patients completed the IN-FOCUS survey. Patients saw a mean of 4 physicians before their FCS diagnosis despite 89% reporting an FCS family history. Patients experience multiple physical, emotional, and cognitive symptoms in addition to FCS-related comorbidities. Notably, 35% of those who answered the survey have experienced acute pancreatitis, averaging 14 lifetime episodes per patient. In the preceding 12 months, 46% of patients had an FCS-related hospitalization, averaging 3 nights’ stay. All respondents restricted fat intake, with 27% following an extremely low-fat diet. Despite this, 100% of patients reported fasting TG levels above the normal range. FCS impacted career choice in nearly all patients (97%) and employment status in all patients who were employed part time, disabled, or homemakers, causing many (> 75%) to choose careers below their level of abilities. Furthermore, 2/3 of patients reported FCS had a significant impact on their decision regarding whether to have children. Most report significant interference with their emotional/mental well-being, social relationships, and the majority were concerned about the long-term impact of FCS on their health (89%).

**Conclusions:**

This study provides the first and largest study to investigate the multi-faceted psychosocial and cognitive impacts of FCS on patients. Canadian patients with FCS experience significant multi-faceted burdens that diminish their quality of life, employment opportunities, social relationships, and mental/emotional well-being. These results highlight the need for greater disease awareness, improved clinical diagnosis, broader clinical management for heterogenous symptoms, and more effective treatment options for FCS.

## Introduction

Familial chylomicronemia syndrome (FCS) is a rare autosomal recessive disorder characterized by persistent, very high elevation of plasma triglyceride (TG), with levels almost always greater than 10 mmol/L (885 mg/dL) [1, 2]. FCS, also known as lipoprotein lipase deficiency (LPLD or type 1 hyperlipoproteinemia), results from homozygosity or compound heterozygosity for null mutations in one or more genes that compromise chylomicron-lipolysis and clearance, most commonly the lipoprotein lipase (*LPL*) gene or, less frequently, other genes directly affecting LPL activity, namely *APOC2*, *APOA5*, *LMF1* and *GPIHBP1*^2^. Affected individuals present with very high TG levels as LPL plays a critical role in the hydrolysis of TG-rich lipoproteins [1]. Fasting plasma TG levels for FCS patients can range from 10 to more than 100 times normal levels [1, 3, 4].

The prevalence of FCS is estimated at approximately 1–2 per million globally, with an increased prevalence in some populations with founder effects including French Canada where the prevalence is approximately 100-fold higher (1:10,000), particularly in the Charlevoix-Saguenay-Lac-Saint-Jean region in Eastern Quebec [5, 6]. Patients with FCS experience a variety of clinical symptoms, with some of the most common being eruptive xanthomas, lipaemia retinalis, recurrent abdominal pain, acute pancreatitis and hepatosplenomegaly. Acute pancreatitis (AP) is the most severe and prevalent complication of FCS affecting ≥50% of patients [7, 8]. Recurrent AP can be complicated by multi-organ consequences, including pancreatic insufficiency, and can eventually lead to death. The pathogenesis of recurrent AP in FCS is not completely understood [9, 10]. Compared to other causes, FCS-induced pancreatitis is considered to be a more severe form of pancreatitis with worse clinical outcomes including organ failure, chronic pancreatitis and pancreatic necrosis [9, 11].

Currently, there is no approved therapy in North America for the treatment of FCS and available TG -lowering agents are not effective [1]. Nineteen Canadian patients have been treated with alipogene tiparovec (Glybera), the first LPL gene replacement therapy, being authorized in occident, but this treatment was not curative [12–14]. The mainstay of symptom management for patients with FCS is still severely restricting dietary fat < 20 g/day, use of medium chain triglycerides (MCT) and avoiding alcohol consumption and specific medications that can increase TG levels [1, 2]. Prolonged compliance with these strict requirements is especially difficult and does not prevent the risk of pancreatitis in all patients [2, 15].

Characterization of the holistic burden of FCS has been historically limited in the literature, but the Investigation of Findings and Observations Captured in Burden of Illness Survey in FCS Patients (IN-FOCUS) study has significantly added to that base, describing physical, psychosocial and cognitive symptomology, comorbidities and the resulting impact of these factors on employment and quality of life [12, 16]. These prior publications have characterized the burden of illness for FCS patients globally and specifically, for United States patients with FCS. The current report presents an analysis from the Canadian subset of patients who agreed to participate in IN-FOCUS, which is especially needed with the high frequency of LPL deficiency in certain regions of the country [5].

## Patients and methods

### Study design

IN-FOCUS was a web-based survey conducted in patients diagnosed with FCS. This research characterized the experience of patients living with FCS in a quantitative manner. The survey instrument was developed and refined in consultation with expert physicians, dieticians and patients. The survey also included validated patient reported outcomes (PRO) scales such as the Pancreatitis Quality of Life Instrument and the Short Form (SF) 36 Health Survey. Patient selection and study design are summarized herein and have been detailed elsewhere [11]. Informed written consent was obtained from all participants and the institutional review board of the University of Mississippi approved this study’s protocol and survey instrument.

A list of 41 physical, emotional and cognitive symptoms associated with FCS was developed and refined based on review of published literature and consults with medical experts and patients. Patients were asked to indicate the symptoms they experienced due to FCS in the past 12 months from two perspectives: 1) typical symptoms and 2) symptoms as experienced at their worst or most severe. Patients rated the severity and frequency of their symptoms on a 7-point Likert scale (1 = very mild, 7 = very severe). Patients were also asked to report the impact of FCS on their lives on a Likert scale (range, 1–7; 1 = no interference at all, 7 = significant interference). Patients reported their current employment status and answered a series of questions to assess the extent to which FCS has had an impact on their career choice, employment status, and/or ability to fulfill responsibilities at work. If not currently a student or employed full- or part-time, patients were asked if they had been previously employed.

Survey data were collected between June 24, 2016, and February 24, 2017 from respondents in 10 countries (Australia, Germany, India, Netherlands, Portugal, Spain, Sweden, United Kingdom, Canada, and United States) [15, 16]. The data presented in this manuscript focus on data from Canadian respondents only.

### Patients

Patients were recruited via word of mouth, on-line support/advocacy groups, social media outlets as well as through their physicians. Physicians treating FCS patients were provided with information about this study and subsequently shared this information with their eligible and interested patients.

The following criteria were met by eligible patients: At least 18 years old, have not participated in a clinical trial for an investigational FCS treatment in the previous 6 months, physician diagnosis of FCS, or LPL deficiency or Fredrickson type 1 Hyperlipoproteinemia or high TG level with a history of experiencing pancreatitis or high TG level with a history of severe abdominal pain resulting in hospital admission, fasting TG level ≥ 750 mg/dL (8.4 mmol/L) determined by the most recent fasting TG test or fasting TG level < 750 mg/dL determined by the most recent fasting TG test with patient-reported dieting to limit fat consumption. Additionally, one of the following four conditions had to be met: genetic diagnosis of FCS, family history of FCS or Fredrickson type 1 Hyperlipoproteinemia, history of repeated periods of abdominal pain requiring hospital admission or emergency department visits that were attributed to high TG levels in the absence of another known cause, or patient history of high TG levels in the absence of another known cause.

### Statistical analysis

Analyses were conducted using SPSS Statistics 22 (IBS, Armonk NY). Continuous variables, including rating scales, were analyzed either as medians with ranges or as means and standard deviations. Categorical variables were evaluated descriptively as frequencies and percentages of occurrence for each category.

## Results

### Patient sample

A total of 97 Canadian FCS patients were screened (entered the web-survey and answered ≥1 question); of the 42 patients who met the requirements for study participation, 37 completed the questionnaire. Demographics and baseline characteristics of respondents are summarized in Table 1. The median (range) age of respondents was 33 years (18–56 years), and median (range) age at FCS diagnosis was 9 years (2–19 years). The sample consisted of majority male (89%) respondents.

**Table 1:** Baseline Demographics and Characteristics

	Patients (***n*** = 37)
Males, n (% of total)	33 (89)
Current age, median (range)	33 (18–56)
Age at FCS diagnosis, median (range)	9 (2–19)
Family history of FCS, n (% of total)	33 (89)

### Journey to FCS diagnosis

Patients were seen by an average of 4 physicians before receiving a diagnosis of FCS (Table 2). The most common specialists to make the FCS diagnosis were endocrinologists, pediatricians, and pancreatologists. The most common reasons leading to patients’ FCS diagnosis included experiencing symptoms later attributed to FCS (49%), a family history of FCS (46%) and experiencing hospitalization(s) due to pancreatitis (41%). At diagnosis, all patients fasting TG levels were ≥ 8.4 mmol/L. Patient-reported family history of FCS in this cohort was common, with 89% of patients reporting one or more family members with diagnosed FCS. The most common family members with FCS were the patient’s father (35%), uncle (30%), and paternal grandfather (27%). Ten patients (27%) reported receiving a misdiagnosis before receiving a correct FCS diagnosis, 57% did not remember whether they were misdiagnosed prior to FCS diagnosis, and 16% were not misdiagnosed before FCS diagnosis. Family history did not appear to influence the accuracy of diagnosis as all patients that were misdiagnosed had family members with FCS. In those misdiagnosed, FCS was initially diagnosed as acute pancreatitis of unknown cause (50%), hypertriglyceridemia (40%), and food allergy (10%).

**Table 2:** Path to FCS Diagnosis

	N (%)
Number of physicians seen for symptoms before FCS diagnosis, median (range)	4 (2–6)
**Common reasons leading to diagnosis of FCS**	
Symptoms that I later learned were due to FCS	18 (49)
Family history of FCS	17 (46)
Hospitalization(s) due to pancreatitis	15 (41)
Abnormal lipid levels in routine bloodwork	7 (19)
Colic and/or failure to thrive in infancy	2 (5)
Development of unexplained hepatosplenomegaly	1 (3)
**Family History of FCS**	33 (89)
Father	13 (35)
Uncle	11 (30)
Paternal Grandfather	10 (27)
**Fasting triglyceride levels at diagnosis**	
8.4- < 11.3 mmol/L	26 (70)
11.3- < 14.1 mmol/L	10 (27)
14.1- < 17 mmol/L	1 (3)
**Physician specialty who made FCS diagnosis**	
I don’t know	10 (27)
Endocrinologist	10 (27)
Pediatrician	6 (16)
Pancreatologist	4 (11)
Nephrologist	2 (5)
Primary Care Physician	2 (5)
Dermatologist	1 (3)
Cardiologist	1 (3)
Metabolic Specialist	1 (3)
**Prior to being correctly diagnosed with FCS**	
Patients with misdiagnoses	10 (27)
**Most common misdiagnoses**	
Acute pancreatitis of unknown cause	5 (50)
Hypertriglyceridemia	4 (40)
Food allergy	1 (10)

### Symptomology

The mean number of typical symptoms patients experienced was 5 (median = 4, range: 2–8). The mean number of symptoms patients experienced at their most severe was 4 (median = 4, range: 1–14), with 57% of patients having experienced ≥4 symptoms in their most severe form.

**Fig. 1:**
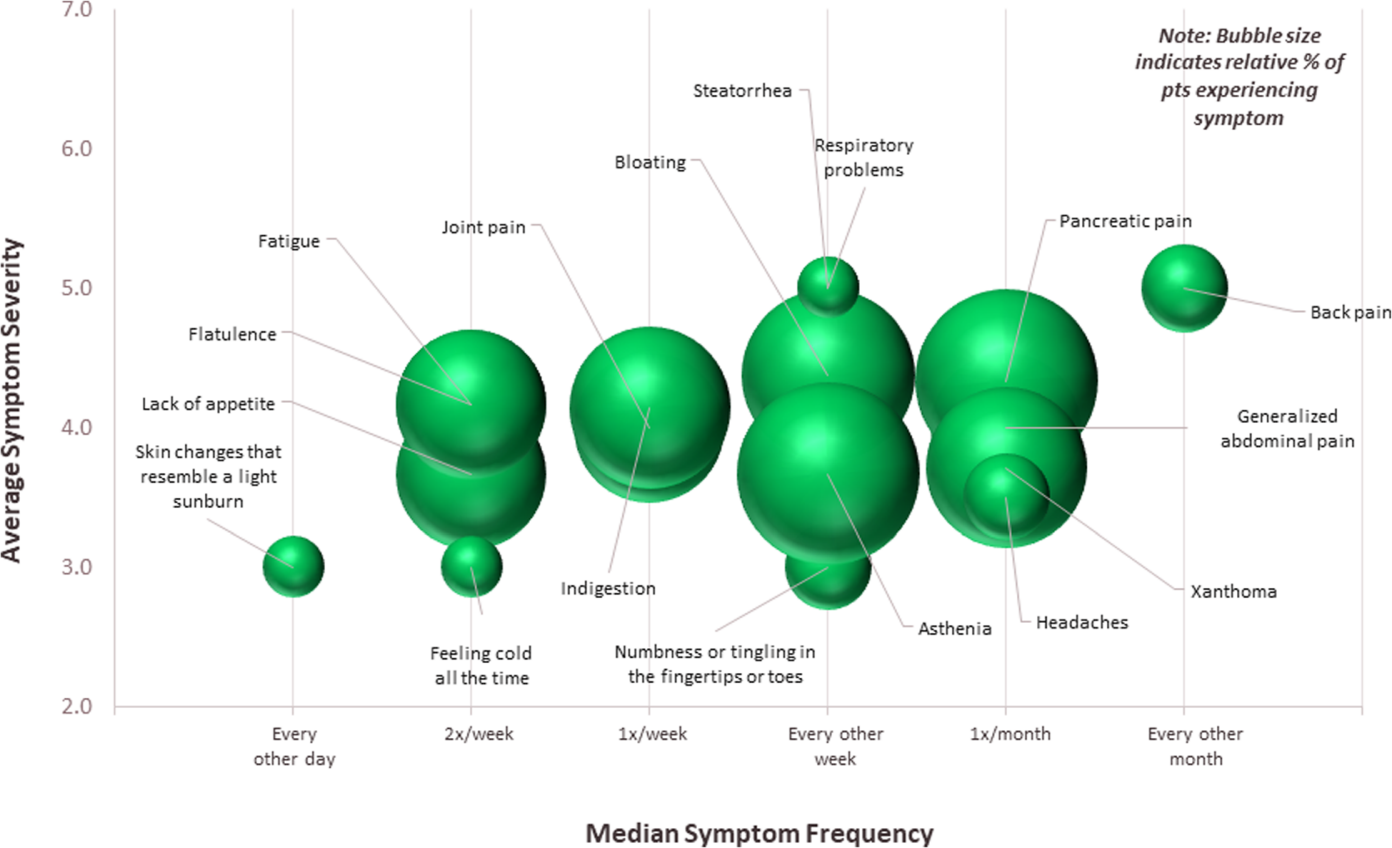
Physical Symptoms at their worst or most severe. For each symptom selected, patients indicated symptom severity and frequency. Severity was recorded on a Likert scale (range, 1–7; 1 = very mild, 7 = very severe). Frequency was recorded by selection from the following options: multiple times per day, daily, every other day, twice a week, once a week, or every other week. Sphere size in the chart is proportional to the percentage of patients who selected each symptom. *Note: Jaundice, experienced less than 1x/year at 5/7 severity is not shown*

**Fig. 2:**
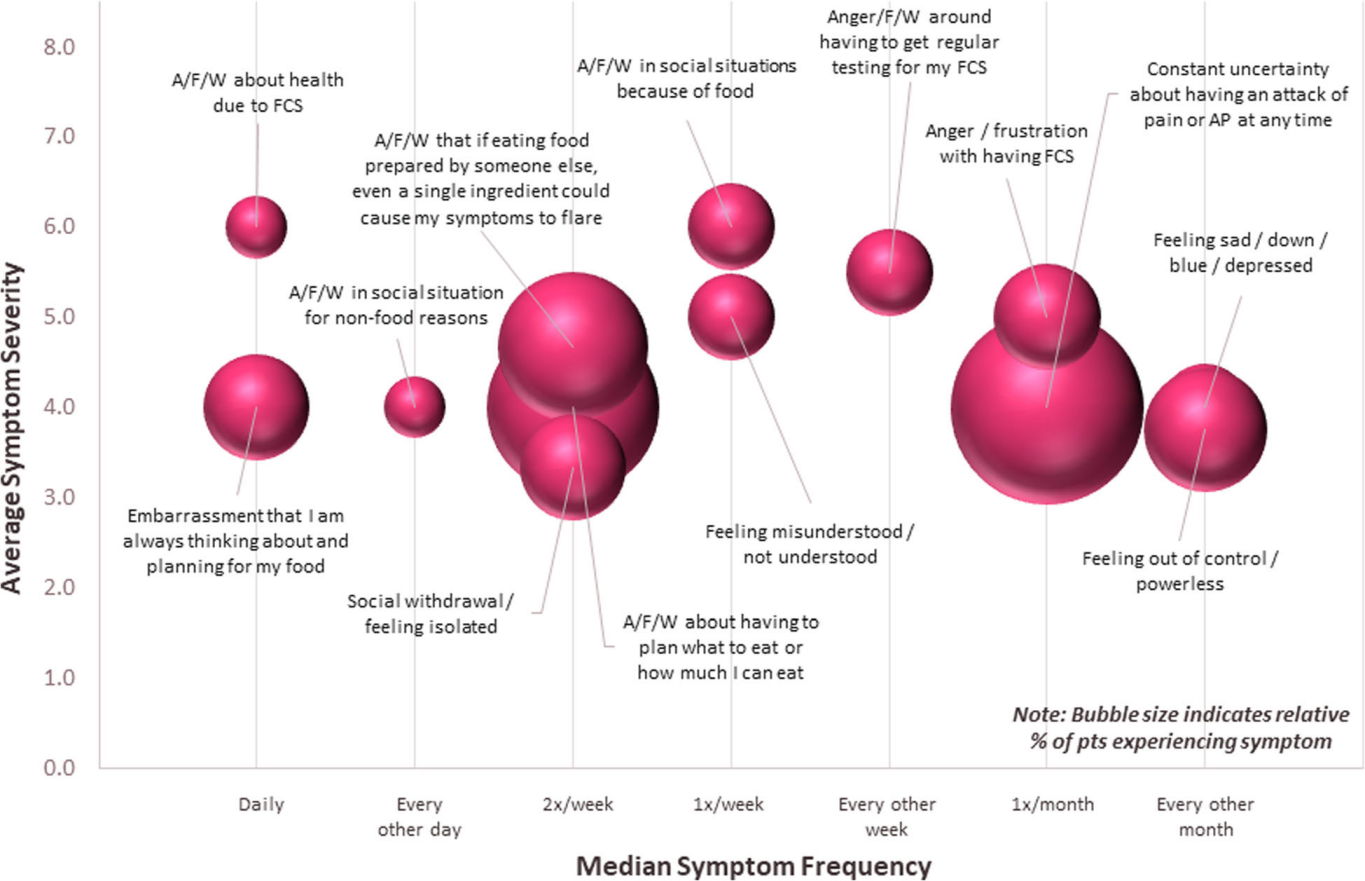
Emotional Symptoms at their worst or most severe. For each symptom selected, patients indicated symptom severity and frequency. Severity was recorded on a Likert scale (range, 1–7; 1 = very mild, 7 = very severe). Frequency was recorded by selection from the following options: multiple times per day, daily, every other day, twice a week, once a week, or every other week. Sphere size in the chart is proportional to the percentage of patients who selected each symptom

**Fig. 3:**
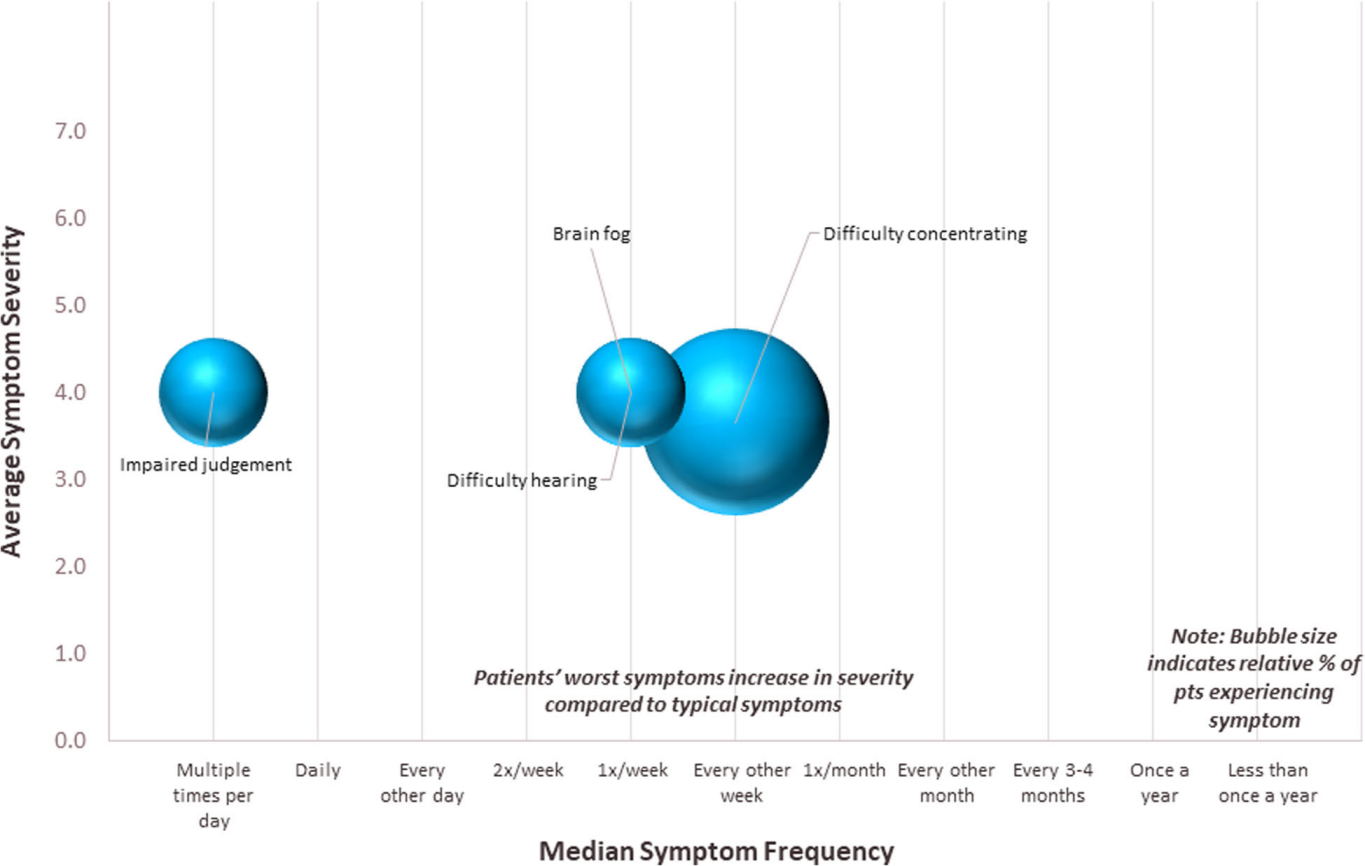
Cognitive Symptoms at their worst or most severe. For each symptom selected, patients indicated symptom severity and frequency. Severity was recorded on a Likert scale (range, 1–7; 1 = very mild, 7 = very severe). Frequency was recorded by selection from the following options: multiple times per day, daily, every other day, twice a week, once a week, or every other week. Sphere size in the chart is proportional to the percentage of patients who selected each symptom

Figures 1, 2 and 3 show the incidence, frequency and severity of physical, emotional, and cognitive symptoms respondents experienced at their worst or most severe, respectively. The size of each sphere in the figures reflects the relative proportion of patients reporting each symptom. The most commonly reported physical symptoms were pancreatic pain (24%), asthenia (24%), bloating (22%), indigestion (19%), and xanthoma (19%) (Table 3). Patients reported experiencing many physical symptoms at a frequency from twice a week to every other week, though some, such as pancreatic pain, were experienced around once a month (Fig. 1). The most commonly reported emotional symptoms were constant uncertainty about having an attack of pain or AP at any time (27%), anxiety/fear/worry about having to plan what to eat or how much to eat (22%), anxiety/fear/worry that if eating food prepared by someone else, even a single ingredient could cause symptoms to flare (16%), and feeling out of control or powerless (11%) (Table 3). Patients reported experiencing many emotional symptoms every other day, but some were experienced less often, around once a month or every other month (Fig. 2). The most commonly reported cognitive symptoms were difficulty concentrating (8%), difficulty hearting (3%), brain fog (3%) and impaired judgement (3%) (Table 3). All reported cognitive symptoms were experienced once per week apart from impaired judgement, which was experienced multiple times per day (Fig. 3).

**Table 3:** Frequency of Symptoms at Most Severe Symptomology: Physical Emotional, and Cognitive

	N (%)
**Physical (21 symptoms)**	
Pancreatic Pain	9 (24)
Asthenia	9 (24)
Bloating	8 (22)
Indigestion	7 (19)
Xanthoma	7 (19)
Generalized abdominal pain	6 (16)
Joint pain	6 (16)
Lack of appetite	6 (16)
Fatigue	6 (16)
Flatulence	6 (16)
Back pain	2 (5)
Numbness or tingling in the fingertips or toes	2 (5)
Headaches	2 (5)
Respiratory problems	1 (3)
Jaundice	1 (3)
Skin changes that resemble a light sunburn	1 (3)
Feeling cold all the time	1 (3)
Steatorrhea	1 (3)
**Emotional (13 symptoms)**	
Constant uncertainty about having an attack of pain or AP at any time	10 (27)
A/F/W about having to plan what to eat or how much I can eat	8 (22)
A/F/W that if eating food prepared by someone else, even a single ingredient could cause my symptoms to flare	6 (16)
Feeling out of control / powerless	4 (11)
Embarrassment that I am always thinking about and planning for my food	3 (8)
Social withdrawal / feeling isolated	3 (8)
Anger / frustration with having FCS	3 (8)
A/F/W in social situations because of food	2 (5)
Feeling sad / down / blue / depressed	2 (5)
Feeling misunderstood / not understood	2 (5)
Anger/F/W around having to get regular testing for my FCS	2 (5)
A/F/W about health due to FCS	1 (3)
A/F/W in social situation for non-food reasons	1 (3)
**Cognitive (7 symptoms)**	
Difficulty concentrating	3 (8)
Difficulty hearing	1 (3)
Brain fog	1 (3)
Impaired judgement	1 (3)

A/F/W, anxiety/fear/worry

Note: Symptoms reported by the Canadian IN-FOCUS cohort shown, for full symptom list, see global manuscript by Davidson et. al, 2018. Common language descriptors were included for symptoms with more technical names (e.g., asthenia described as “feeling of physical weakness”)

### Comorbidities

**Fig. 4:**
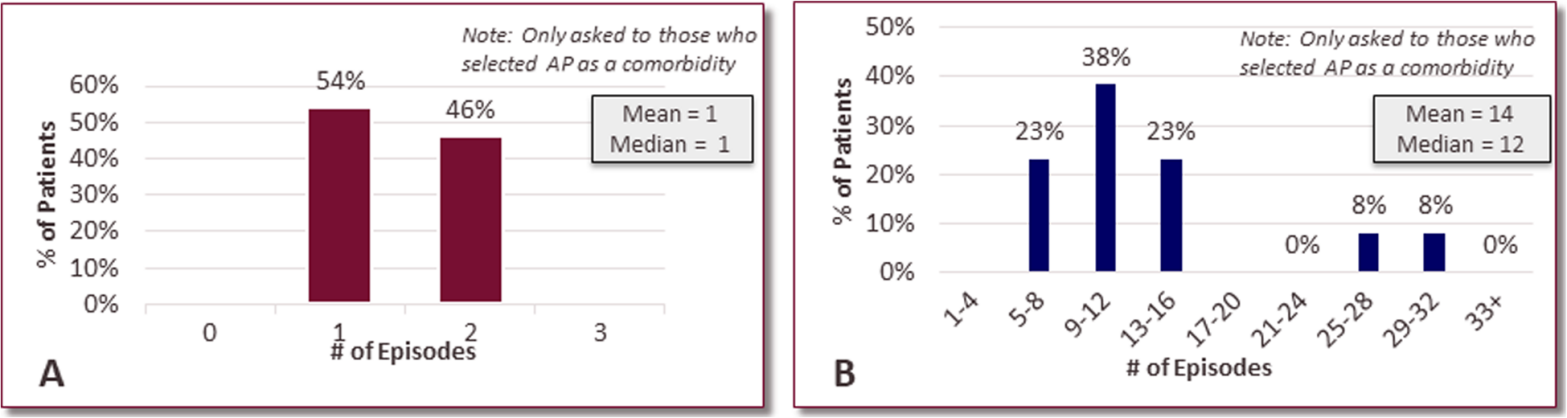
Experiences with acute pancreatitis. Number of acute pancreatitis episodes experienced by patients with FCS, who reported acute pancreatitis as a comorbidity in the past 12 months (**a**) and lifetime (**b**)

Most patients (84%) with FCS reported at least one comorbidity. The most common current comorbidities reported were AP (35%), hypertension (19%), addiction to/dependence on pain medication (14%), eating disorder (14%), and diabetes caused as a complication of FCS (11%) (Table 4). The thirteen (35%) FCS patients that have experienced AP in their lifetime have endured an average of 14 episodes. All of these patients have experienced 1–2 episodes in the past year (Fig. 4a) and two have experienced > 25 AP episodes in their lifetime (Fig. 4b).

**Table 4:** Top Comorbidities Due to FCS

	N (%)
Acute Pancreatitis	13 (35)
Hypertension	7 (19)
Addiction to, or dependence on, pain medication	5 (14)
Eating Disorder (e.g. bulimia, anorexia)	5 (14)
Diabetes, caused as a complication of FCS	4 (11)
Chronic Pancreatitis	3 (8)
Pancreatic Calcification	2 (5)
Peripheral Neuropathy	2 (5)
Splenomegaly	1 (3)
None of the above	6 (16)

### Experiences with acute pancreatitis

All patients that have experienced AP have been hospitalized for at least one episode (data not shown). Patients having experienced AP reported a mean of 7 outpatient and 4 inpatient lifetime hospitalizations due to AP. Two patients (15% of those having experienced AP) were readmitted to a hospital within 30 days of being discharged for AP.

### Health care resource utilization

**Fig. 5:**
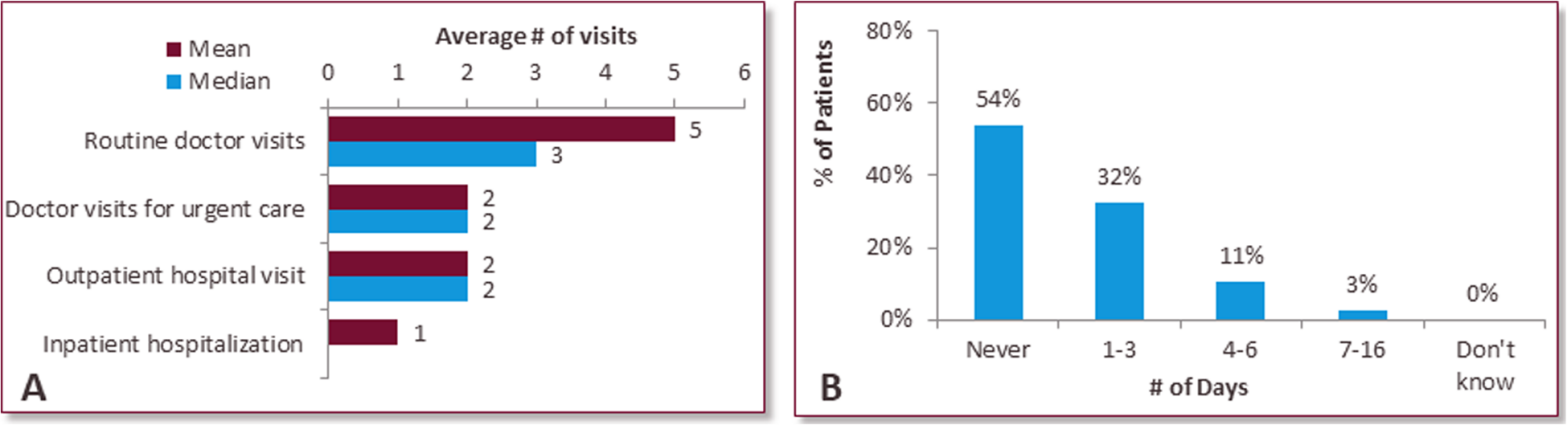
Visits to the doctor/hospital. Average number of doctor / hospital visits for FCS patients (**a**) and average number of days per hospitalization (**b**)

FCS patients reported visiting the doctor an average of 5 times for routine visits, twice for urgent care and outpatient hospital visits and once for inpatient hospitalization in the preceding 12 months (Fig. 5a). 46% of patients reported being hospitalized due to FCS in the preceding 12 months, with an average hospital stay length of 3 nights (Fig. 5b).

### Management of FCS

**Fig. 6:**
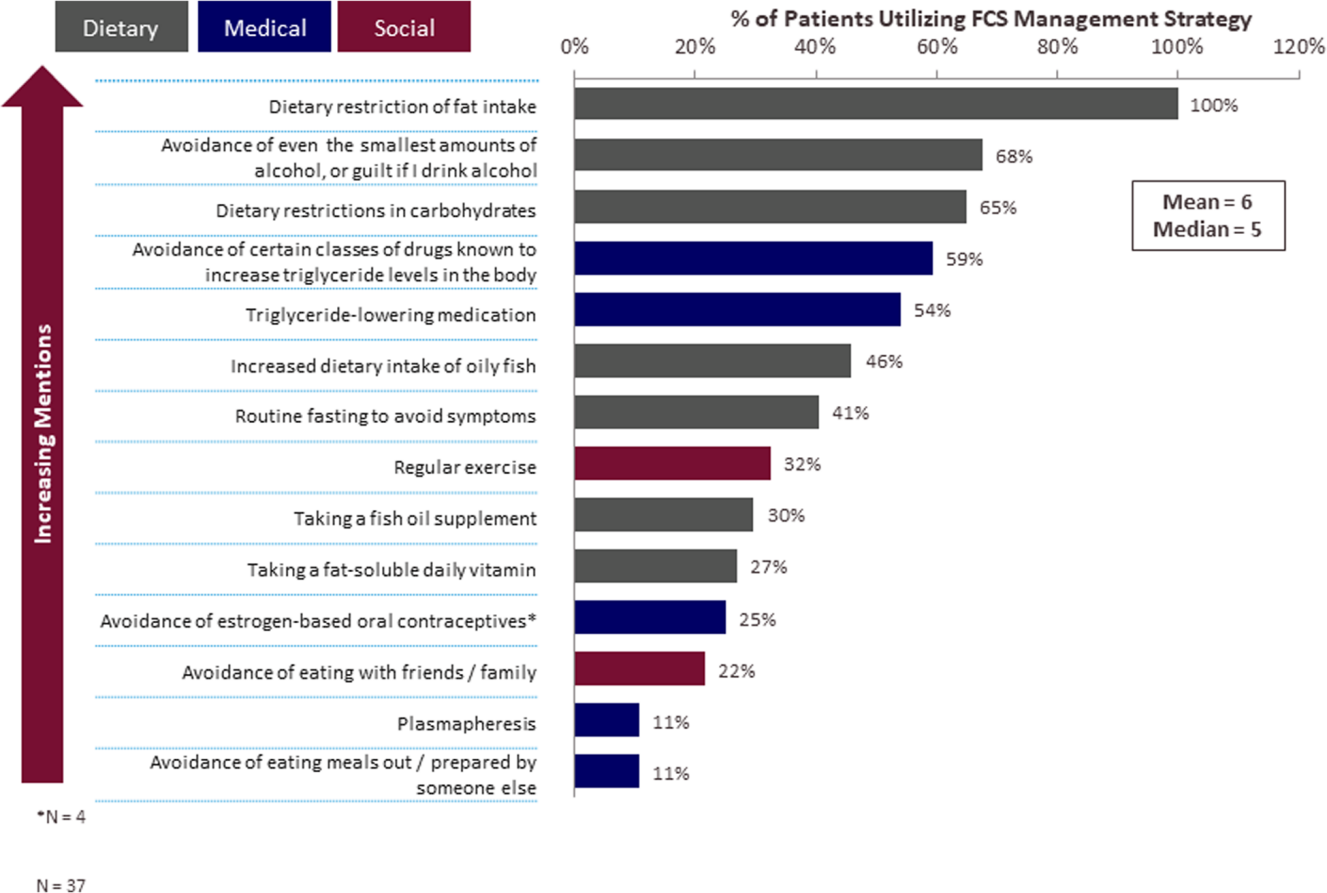
Utilization of FCS Management Strategies. Note: Because patients may use > 1 strategy, responses sum to > 100%

FCS patients reported utilizing an average of 6 distinct strategies to manage their FCS (Fig. 6). All patients reported restricting their dietary fat intake to ≤30 g per day. However, only 27% of patients reported following an extremely low-fat diet, adhering to levels ≤20 g (data not shown). Many patients avoided alcohol (68%), restricted their consumption of carbohydrates (65%) and over 40% of patients routinely fasted to mitigate their symptoms. Despite adherence to this restrictive diet, 100% of the patients reported current TG equal to or greater than 5.6 mmol/L (Table 5). The majority of patients reported that managing their symptoms was extremely time-consuming (86%) and energy-draining (78%) and their current approach to managing FCS symptoms was rigid and prohibitive (78%) (Supplemental Figure 1). All FCS patients reported that trying to limit/manage daily fat intake was challenging to some degree [5–7 rating on a Likert scale; range, 1–7; 1 = very easy, 7 = very challenging].

**Table 5:** Fasting Triglyceride Levels at Most Recent Test

	N (%)
5.6- < 8.4 mmol/L	25 (68)
8.4- < 11.3 mmol/L	12 (32)

### Impact of the disease on personal, social and professional life

**Fig. 7:**
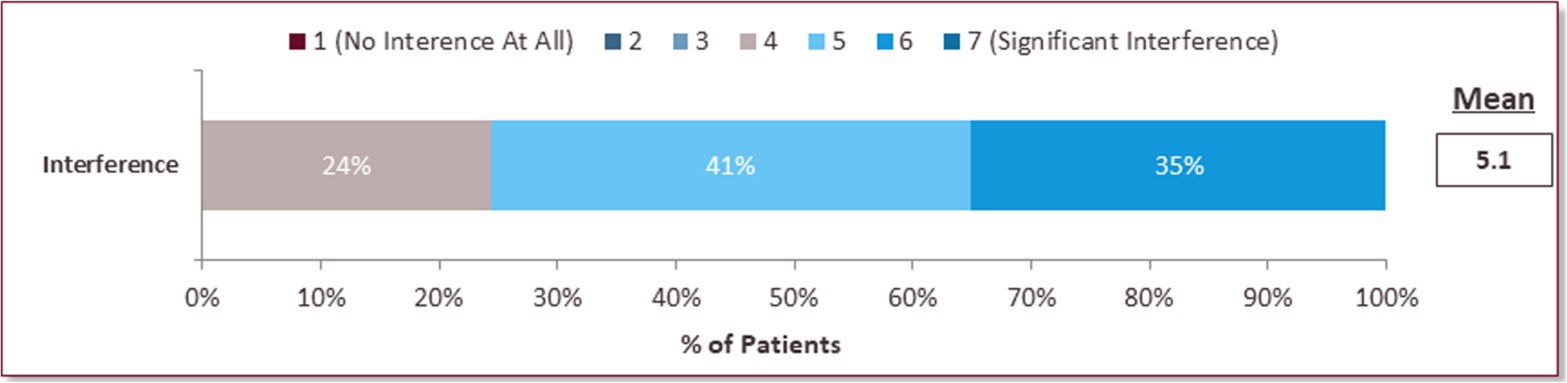
Impact of FCS on Patients’ Lives

**Fig. 8:**
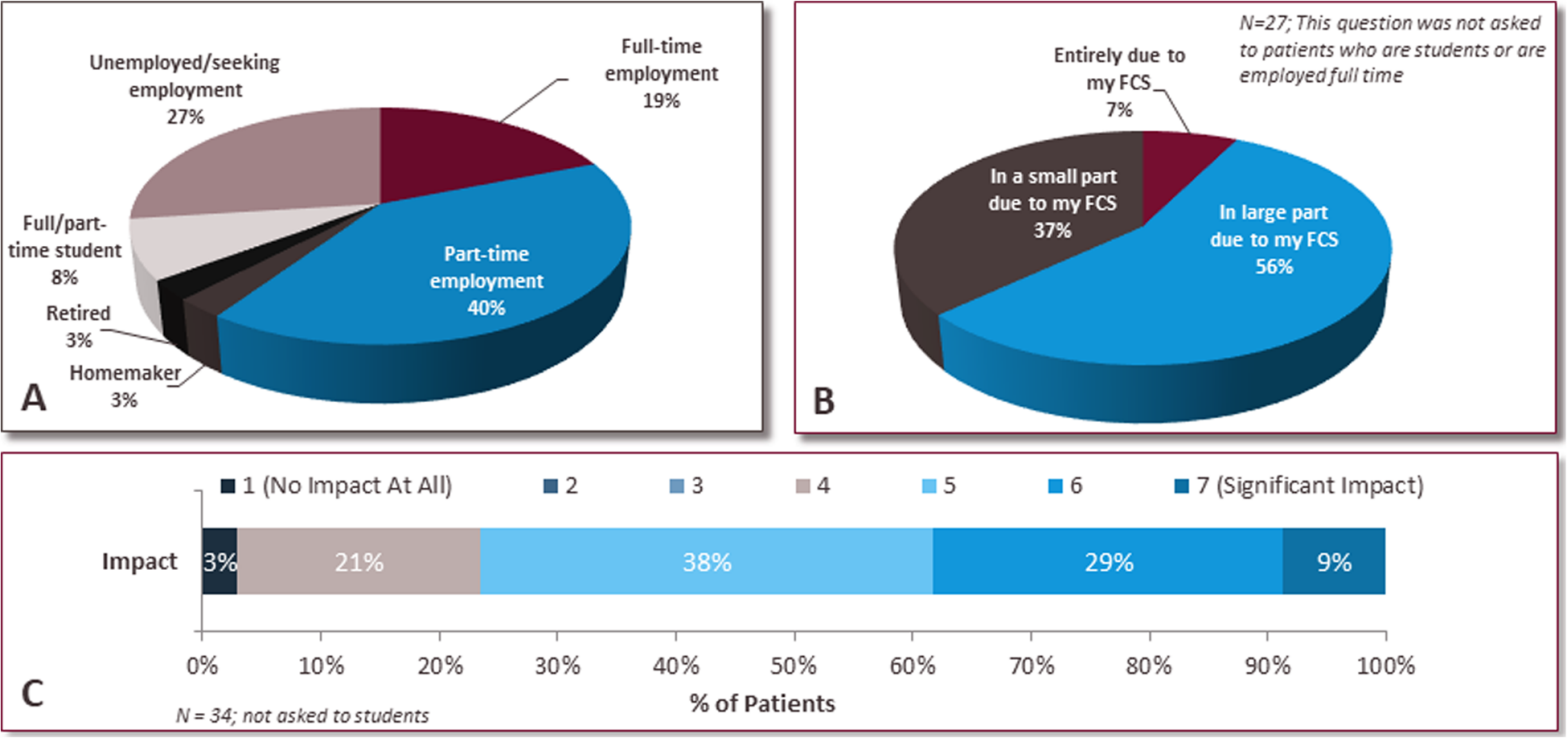
Impact of FCS on employment status. All patients indicated their current employment status (**a**), and patients, excluding students and full time employed patients, indicated the impact of FCS on employment status (**b**). Patients excluding students indicated the overall interference of FCS on career choice (**c**)

All patients reported that FCS interferes with their lives, with the majority (76%) indicating a moderate to significant impact (5–7 rating) (Fig. 7). Current employment status is shown in Fig. 8a. Many patients (63%) who were employed part-time, disabled or homemakers reported that their current employment status was in large part or entirely influenced by their FCS (Fig. 8b). The majority of patients (97%) who were not students, reported that their career choice was in some part impacted by their FCS (Fig. 8c). More than 80% of patients chose careers that required less travel, were less demanding and believed that their ideal career was not conducive to adhering to a strict diet. 76% of patients purposely chose a career below their level of abilities when they are well due to their FCS (Supplemental Figure 2). Among the patients who worked full-time or part-time, 97% reported FCS has impacted their ability to fulfill their responsibilities at work to some degree and 55% have taken time off due to their FCS in the past 12 months (data not shown). These FCS patients reported having to take an average of 14 days off from work specifically due to their FCS.

**Fig. 9:**
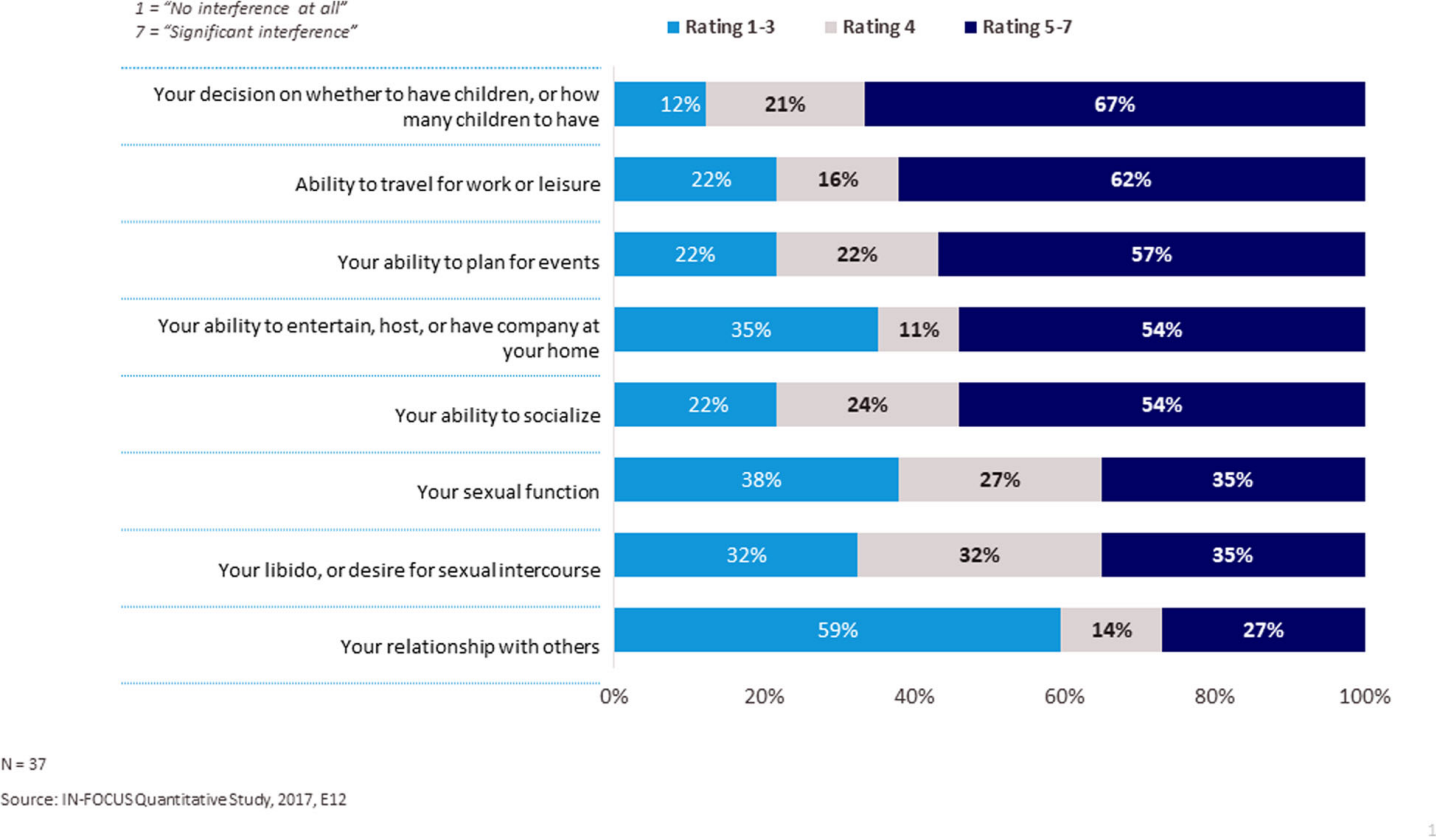
Impact on Social Relationships and Activities

Patients reported that the greatest negative impact FCS has on their social relationships and activities were surrounding decisions to have children (67%), and their ability to travel for work or leisure (62%) (Fig. 9). Most patients reported that FCS significantly interfered with their emotional well-being (81%), stress and anxiety (78%), feeling of self-worth (78%), mental ability (76%) and quality of sleep (73%) (Supplemental Figure 3). The majority (> 80%) of patients were concerned about the long-term impact of FCS on their health and other aspects of their lives such as their ability to live a normal life and losing their job (Supplemental Figure 4). 70% of patients experienced a significant financial impact from their FCS specifically because maintaining a low-fat diet and purchasing food that adheres to their strict diet was expensive. FCS patients additionally reported they had avoided spending money on other things, so they could afford their low-fat diet (68%). Despite that, almost two in three (65%) reported that they have been unable to make some lifestyle changes recommended to them because of the cost associated with doing so (Supplemental Figure 5).

## Discussion

This research confirms the burden experienced by Canadian patients with FCS through characterization of the difficult journey to diagnosis and the multidimensional symptoms and comorbidities attributed to FCS that interfere with personal, social and professional life. This study aimed to capture a comprehensive picture of the patient experience through holistically assessing the impact on patients’ quality of life. The contribution of Canadian patients to the IN-FOCUS is globally significant given the prevalence of FCS among French Canadians from eastern Quebec [5]. The high prevalence of FCS in this population is due to a founder effect and is a consequence of a phenomenon called endogamia [17]. At the molecular level, a majority of French Canadians with FCS are homozygous or compound heterozygous for proven null LPL gene variants associated with low post-heparin LPL activity, particularly the p.Pro234Leu and p.Gly215Glu variants. The findings presented here provide a Canadian patient perspective of the burden of FCS, building upon findings reported elsewhere from the global sample and other sub-cohorts [15, 16]. Although the sample size is small, results from the Canadian cohort of IN-FOCUS are largely consistent with themes from the global study, reflecting that the Canadian cohort is a relatively representative subset of the IN-FOCUS study.

The data clearly demonstrate that the path to FCS diagnosis is lengthy and can often be convoluted with many challenges [18, 19]. Often, basic clinical variables that should serve as red flags are not used in the diagnostic work-up. For example, despite the large majority of patients having a family history of FCS and the eventual attribution of FCS diagnosis to this family history in nearly half, patients cycled through an average of 4 physicians before being correctly diagnosed with FCS. Many patients in this survey (41%) experienced at least one acute pancreatitis-associated hospitalization prior to FCS diagnosis, underscoring the danger that delayed diagnosis poses to patients’ lives. The rarity of the disease along with the heterogeneous nature of FCS and the lack of consensus guidance on diagnostic criteria make it unsurprising that physicians who encounter FCS may not be well versed in its diagnosis. The Canadian FCS founder population and the challenge raised by the existence of multifactorial, late-onset and treatable forms of recurrent chylomicronemia underscore the important need to increase education to improve FCS awareness and knowledge within the Canadian physician community and ultimately reduce delays in diagnosis [5, 20].

Patients with FCS experience an average of 5 clinical signs or symptoms typically, and 4 symptoms when symptoms are at their worst or most severe. Specific symptoms vary widely, as illustrated by the 38 FCS symptoms experienced by these 37 patients across physical, emotional and cognitive domains. The average number of symptoms patients experience when their FCS is at its worst may be numerically lower or no different than the number of their typical symptoms, but worst symptoms stand out, delineated by the greater intensity and/or frequency. Beyond physical and cognitive symptoms, this research uncovers the emotional burden patients face, particularly with respect to the uncertainty of when symptoms will occur, the fear of AP and the stress of adhering to a highly restrictive diet.

Patients with FCS commonly experience comorbidities, with AP being the most frequently reported. In this sample over one-third of patients reported experiencing AP. In a study in a similar population, 54% of adult Canadian FCS patients had experienced at least one episode of AP [21]. AP can significantly inhibit patients physically while exacerbating the emotional burden and stress caused by their disease. Further, the combination of severe FCS physical symptoms and associated comorbidities can impact patients’ healthcare resource utilization and FCS’ burden on the healthcare system. This is demonstrated by the significant portion of patients in this research (46%) reporting one or more hospitalization in the preceding 12 months directly related to FCS, the multiple nights’ stay associated with each of these hospitalizations, and the additional multiple FCS-related visits for urgent and routine care. All patients are taking steps to manage their FCS by restricting their fat intake, with 27% following an extremely restrictive low-fat diet (≤20 g/day). Despite these efforts, 100% of patients reported TG levels above the normal range. This highlights both the difficulty in following the low-fat diet recommended for FCS, and the lack of effectiveness such adherence has had on lowering TG levels in patients who are able to follow such a restrictive low-fat diet. Cost and outcome modeling has suggested a correlation between reduction in TG levels and a reduction in morbidity and mortality associated with costly acute pancreatitis events, underscoring the significant benefit an effective therapy for the disease could have [22]. As evidenced by the current FCS management strategies that are challenging and minimally effective, there is high unmet need for an effective treatment for FCS in Canada.

The persistent and overwhelming burden of symptoms and comorbidities significantly impacts patients’ career choice, long term health, and outlook on life. FCS influences career choice in nearly all patients (97%), causing many (> 75%) to purposely choose careers below their level of abilities that are less demanding and require less travel, due to belief that their ideal career is not conducive to adhering to a strict diet. This suggests patients with FCS may not feel fulfilled by their careers if they are settling for careers that do not challenge or excite them due to their disease. The influence of FCS on patients’ careers and ability to work signals the potential cumulative lifetime financial impact of the disease on earning potential and finances, affecting both patients and their families. Despite settling for a job that is below their abilities, the majority of patients (82%) have concerns about losing their job due to their FCS.

Concerns around the ability to live a normal life and the long-term impact of FCS on health are also shared by the majority of patients with FCS (> 80%), revealing the impact on patients’ life outlook. Strategies for managing FCS are time-consuming, rigid and prohibitive, but patients with FCS also appear to be resilient and have adjusted their lives in order to manage their symptoms. Over half of sampled patients with FCS (59%) are employed full or part-time, despite many indicating that FCS has had a large impact on their employment status, underscoring the determination of these patients.

### Limitations

There are several limitations that should be considered in this report. The sample size of this report, albeit in a rare disease, is limited is thus not representative of the full Canadian population affected from this mendelian trait. Thus, drawing definitive conclusions should be approached with caution, although the convergence with data collected elsewhere is strong. The survey responses were self-reported, meaning results cannot be verified and may be subject to recall bias. The nature of an online survey suggests the potential for selection bias favoring respondents that are younger, more technologically knowledgeable, or have more severe FCS, are comfortable sharing personal health-related experiences, and/or who want their voices heard. Because recruitment of patients was conducted through patient advocacy groups and social media, this study sample represents only a subset of Canadian patients. Furthermore, this analysis represents Canadian survey respondents only and may not represent patients with FCS in other countries. The sample of patients surveyed is majority male (89%), which may have influenced the experiences reported by patients in this survey.

## Conclusions

In conclusion, this report encompassing the experiences of 37 Canadian patients with FCS, reflects the patient perspective on the multidimensional impact FCS has on patients’ lives which is consistent with what has been assessed in other countries. By characterizing symptomology, comorbidities, path to diagnosis, and influence on elements such as social relationships and activities, diet management, mental health, and career choice, among others, a comprehensive picture of the deep impact FCS has on patients emerges. These results highlight the need for greater disease awareness, improved clinical diagnosis, broader clinical management for heterogenous symptoms, and more effective treatment options for FCS. Insights from this research should be used to inform healthcare providers in Canada of the implications of FCS on patients’ lives, and the individualized nature of the FCS patient experience to inform disease management. Further research is warranted to identify systemized avenues for providers to efficiently diagnose FCS, and to elucidate effective treatment strategies.

## Supplementary information

**Additional file 1: Supplemental Figure 1.** Perception of FCS Management Strategies.

**Additional file 2: Supplemental Figure 2.** FCS Influence on Career Choice.

**Additional file 3: Supplemental Figure 3.**: Mental/Emotional Well-being.

**Additional file 4: Supplemental Figure 4.** Impact on Future Outlook.

**Additional file 5: Supplemental Figure 5.** Financial Impact of FCS Associated with Dietary Modifications.

## Data Availability

Not applicable.

## Abbreviations

FCS: Familial chylomicronemia syndrome
TG: triglyceride
LPLD: Lipoprotein lipase deficiency
LPL: Lipoprotein lipase
AP: Acute pancreatitis
MCT: Medium chain triglycerides
IN-FOCUS: Investigation of Findings and Observations Captured in Burden of Illness Survey in FCS Patients
PRO: Patient reported outcomes
SF: Short form
