# Supplementary figures and images for: The burden of familial chylomicronemia syndrome in Canadian patients
Source: Lipids Health Dis. 2020 Jun 2;19:120. doi: 10.1186/s12944-020-01302-x (PMC7268343; doi:10.1186/s12944-020-01302-x)

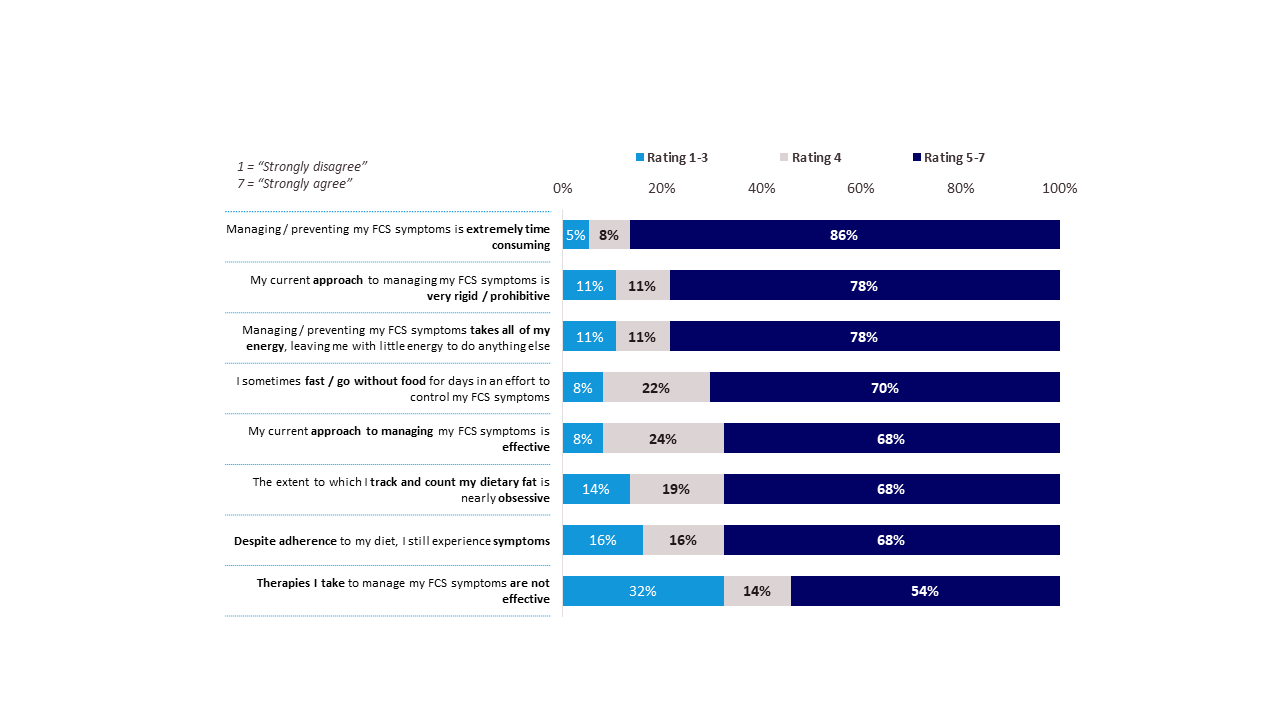

Supplement: Supplementary file 1 — Additional file 1: Supplemental Figure 1. Perception of FCS Management Strategies. [file 12944_2020_1302_MOESM1_ESM.tif]

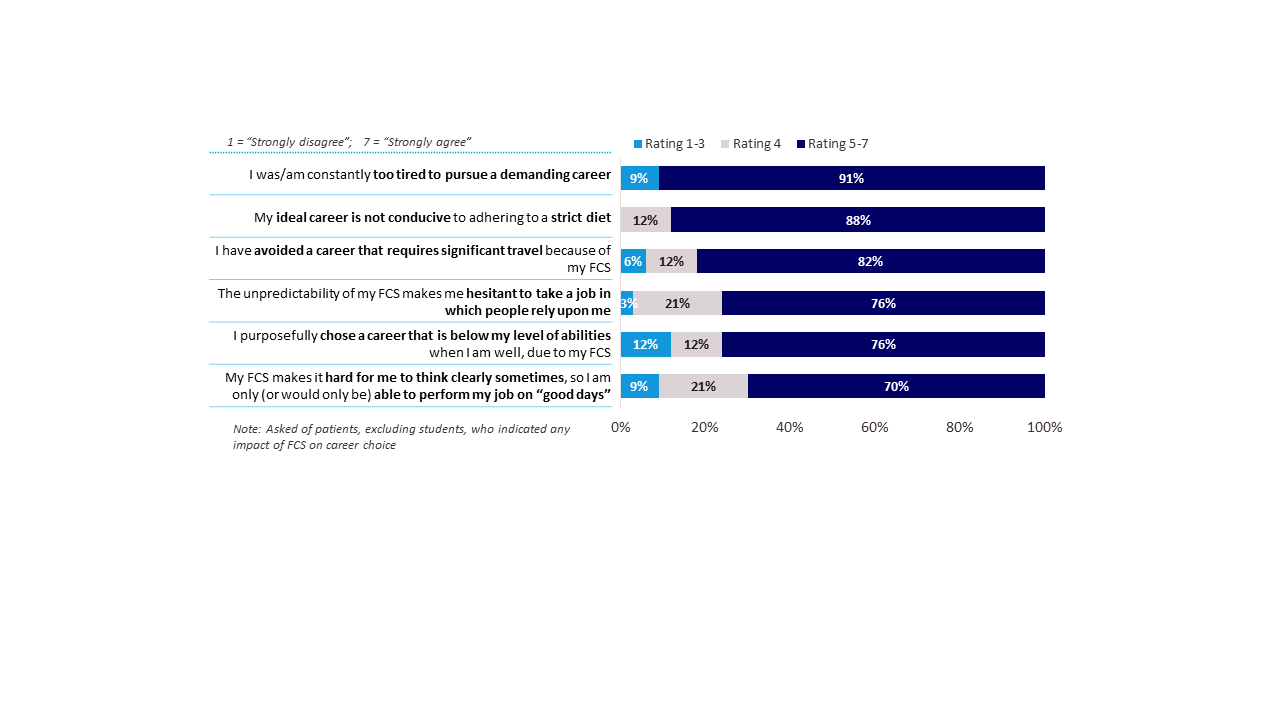

Supplement: Supplementary file 2 — Additional file 2: Supplemental Figure 2. FCS Influence on Career Choice. [file 12944_2020_1302_MOESM2_ESM.tif]

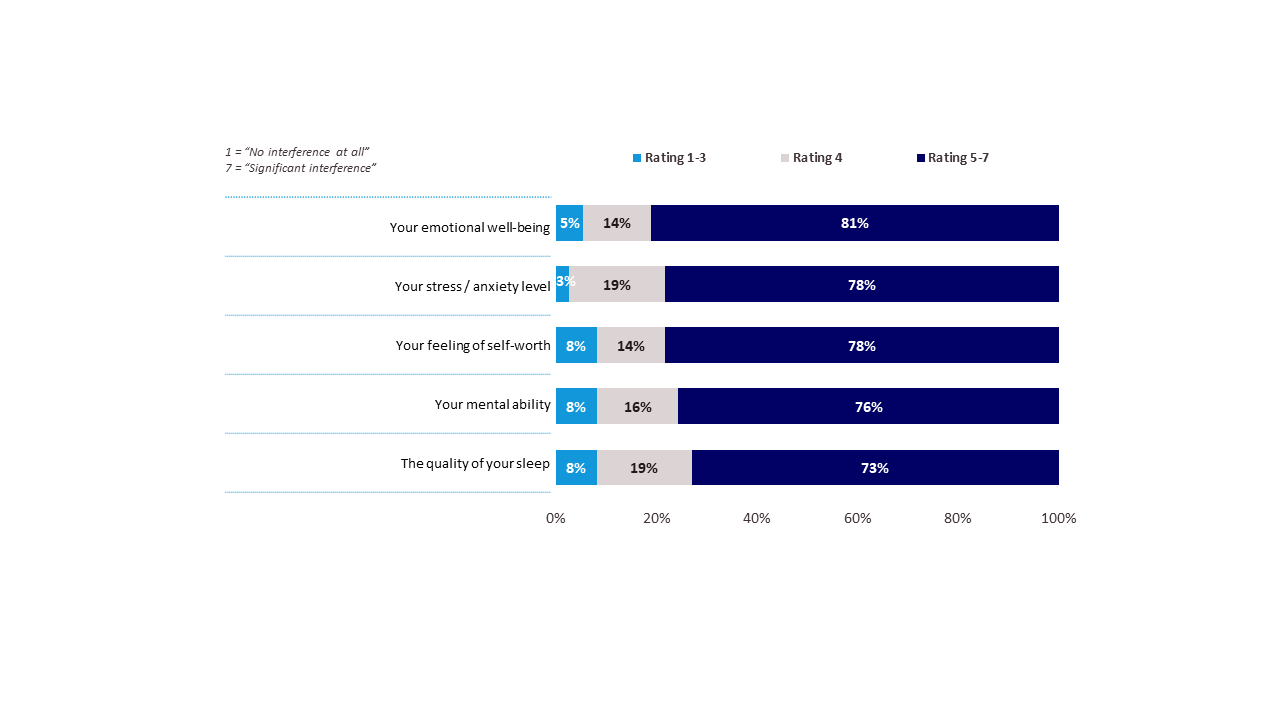

Supplement: Supplementary file 3 — Additional file 3: Supplemental Figure 3.: Mental/Emotional Well-being. [file 12944_2020_1302_MOESM3_ESM.tif]

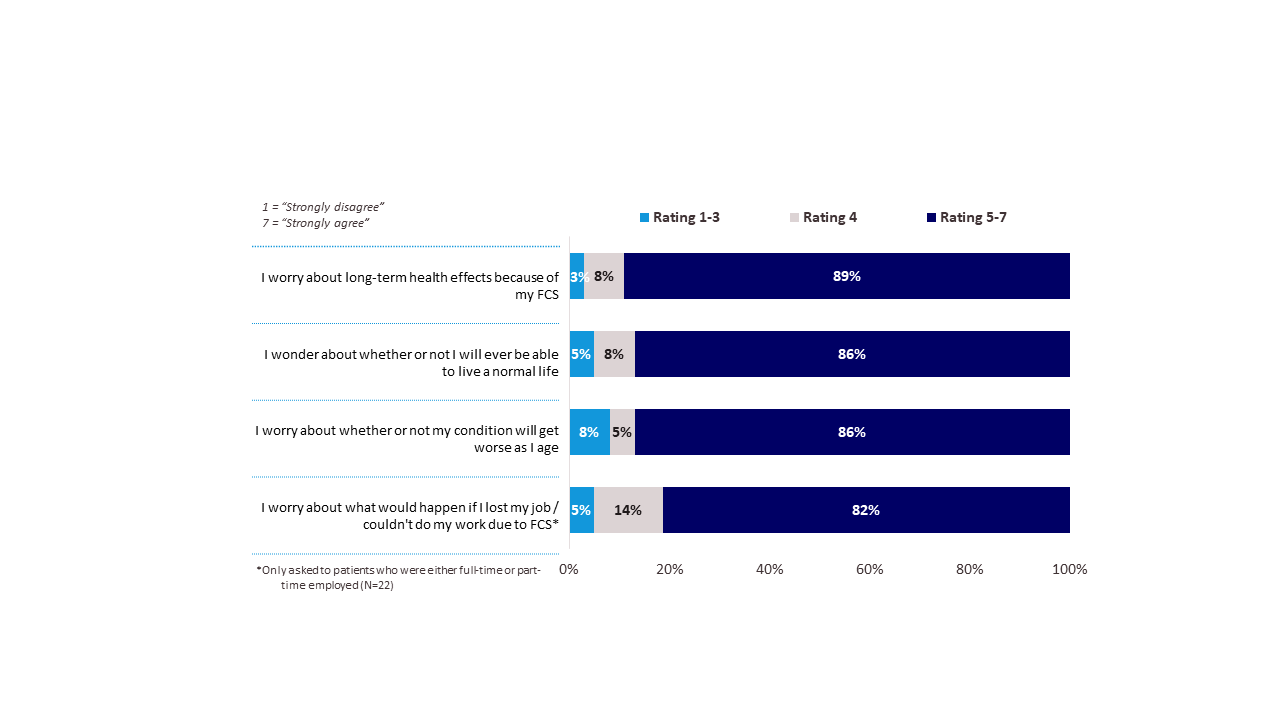

Supplement: Supplementary file 4 — Additional file 4: Supplemental Figure 4. Impact on Future Outlook. [file 12944_2020_1302_MOESM4_ESM.tif]

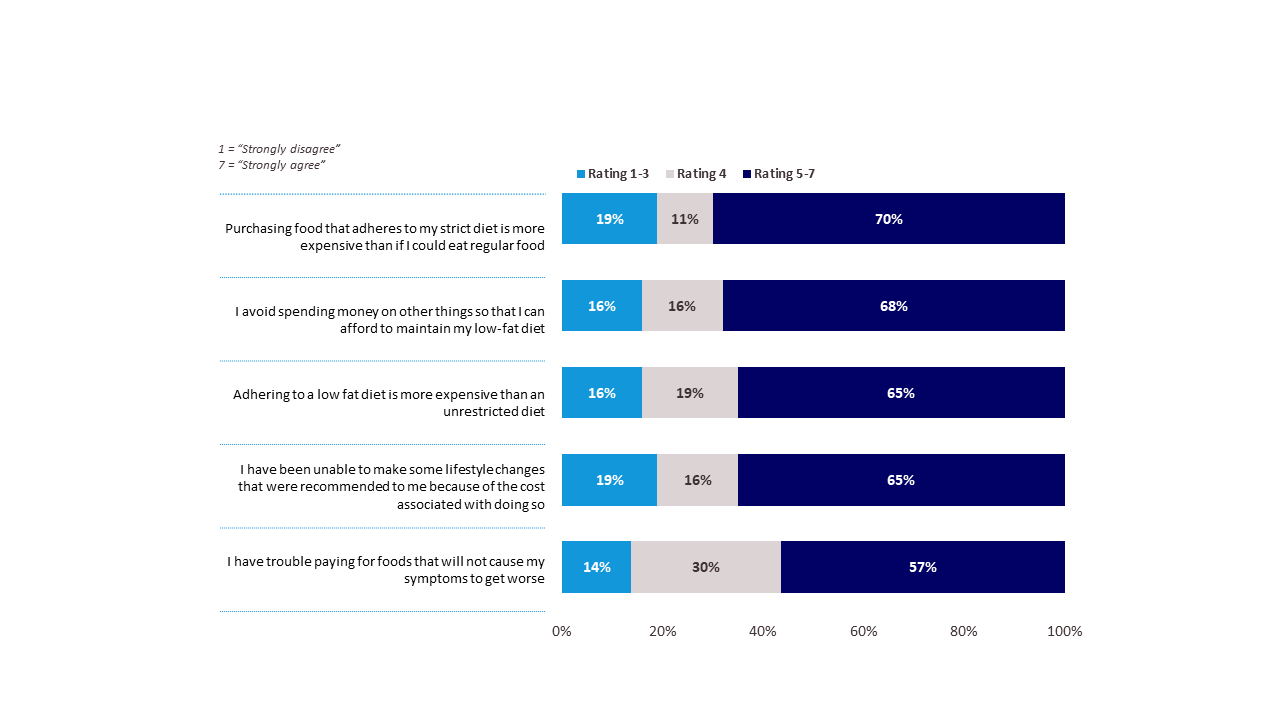

Supplement: Supplementary file 5 — Additional file 5: Supplemental Figure 5. Financial Impact of FCS Associated with Dietary Modifications. [file 12944_2020_1302_MOESM5_ESM.tif]
